# Short-Term Anticoagulation After Cardioversion in New-Onset Atrial Fibrillation and Low Thromboembolic Risk: A Real-World International Investigation

**DOI:** 10.3390/medicina61071200

**Published:** 2025-06-30

**Authors:** Alan Poggio, Andrew P. Sullivan, Lorenzo Rampa, Jason G. Andrade, Matteo Anselmino

**Affiliations:** 1 Department of Clinical and Biological Sciences, MedInTO—Medicine and Surgery, University of Turin, 10125 Turin, Italy; alan.poggio@edu.unito.it; 2 UBC Division of Cardiology, Gordon & Leslie Diamond Health Care Centre, 2775 Laurel St., 9th Floor, Vancouver, BC V5Z 1M9, Canada; apsullivan@mun.ca (A.P.S.); jason.andrade@vch.ca (J.G.A.); 3 Department of Arrhythmology and Electrophysiology, IRCCS Ospedale San Raffaele, 20132 Milan, Italy; rampa.lorenzo@hsr.it; 4 Division of Cardiology, Department of Medical Sciences, “Città della Salute e della Scienza di Torino” Hospital, University of Turin, 10125 Turin, Italy

**Keywords:** atrial fibrillation, cardioversion, short-term oral anticoagulation, thromboembolism, stroke, bleeding

## Abstract

*Background and Objectives*: International guidelines differ on short-term (4-week) oral anticoagulation (OAC) indication after acute cardioversion for recent-onset atrial fibrillation (AF < 12–48 h) in low-risk patients (CHA_2_DS_2_-VA = 0). While Canadian and Chinese guidelines recommend OAC for all, European, Australian and New Zealand, and American guidelines state that such treatment is optional due to the absence of high-quality evidence supporting its indication in this specific scenario. This study aimed to assess physicians’ management of a simple clinical case at an international level, focusing on how they balance ischemic and bleeding risks in a setting lacking any strong evidence-based recommendations. *Materials and Methods*: Six different AF guidelines were evaluated regarding the recommendation for and scientific evidence justifying short-term OAC in this specific setting. Following review, an international questionnaire was developed with *Google Forms 2024* (Mountain View, CA, USA) and circulated among physicians working in the fields of cardiology, internal medicine, intensive care unit, geriatrics, and emergency medicine at 17 centres in Italy, France, and Canada. *Results*: A total of 78 responses were obtained. Younger physicians and cardiologists appeared to administer OAC more frequently compared to older physicians or those working in other specialties (95% CI Fisher’s Exact Test *p* = 0.049 and 0.029, respectively). Significant differences were observed in the use of periprocedural imaging, with transoesophageal echocardiogram (TOE) prior to cardioversion being performed more often in Europe vs. Canada (*p* = 0.006) and in long-term rhythm control, with first-line pulmonary vein isolation (PVI) being offered more frequently by European cardiologists (*p* = 0.013). No statistically significant association was found regarding guideline adherence for OAC administration (*p* = 0.120). *Conclusions*: The real-world antithrombotic management of low-risk (CHA_2_DS_2_-VA = 0), acutely cardioverted AF patients varies significantly among different healthcare systems. Particularly in cardiology departments, reducing the time limit for safely not prescribing OAC to < 12 h, ensuring local access to direct oral anticoagulants (DOACs) and considering regional stroke risk profiles, as well as actively preventing haemorrhage in patients receiving short-term OAC could all limit cardioversion-related complications in this low-risk population.

## 1. Introduction

With an estimated 60 million people affected globally and a steadily rising prevalence, atrial fibrillation (AF) is unquestionably a 21st-century pandemic [1]. As AF is associated with an increased risk of systemic thromboembolism and reduced quality of life, it represents a significant burden to healthcare systems around the world [1]. Cardioversion is often the initial strategy for rhythm control in patients with newly diagnosed acute (<48 h duration) or paroxysmal AF [2]. Direct current electrical cardioversion and pharmacological cardioversion are both commonly used methods to restore sinus rhythm. While electrical cardioversion generally achieves higher success rates (80–90%), pharmacological cardioversion is only successful in about 50% of cases [3]. However, electrical cardioversion requires sedation and may pose more risks in hemodynamically unstable patients [3]. A common complication following successful cardioversion is “atrial stunning,” a transient mechanical dysfunction in atrial contractility seen in 30–80% of patients regardless of the cardioversion method employed [4]. Atrial stunning can even occur after spontaneous conversion to sinus rhythm and its severity is influenced by the AF episode duration, atrial size, and the presence of structural heart disease [5]. Transient diminished atrial contractility is thought to contribute to an increased risk of thromboembolic complications (TECs) in the post-cardioversion period, potentially leading to severe disability, morbidity, or death [5,6,7].

At the global level, the management of patients with acute AF at low objective risk of stroke differs substantially. Considering risk assessment, subjects without any risk factors for thromboembolism (i.e., with CHA_2_DS_2_-VA score equal to 0 [C: Congestive heart failure; H: Hypertension; A_2_: Age 75 or older (2 points); D: Diabetes mellitus; S_2_: Stroke or transient ischemic attack (2 points); V: Vascular disease; A: Age 65 to 74]) are believed to be at low risk of peri-cardioversion TECs if their AF episode duration is <12 h (American, Canadian, and Chinese guidelines indications [8,9,10]), <24 h (ESC 2024 recommendations [11]), or <48 h (according to the British and Australian and New Zealand guidelines on AF [12,13]). While the Canadian and Chinese guidelines recommend short-term oral anticoagulation (OAC) for 4 weeks in all patients after acute cardioversion (regardless of arrhythmia duration or risk factors for TECs) [9,10], the European, U.S., Australian and New Zealand, and UK (NICE) guidelines state that postprocedural anticoagulation is discretionary due to the lack of high-quality evidence supporting such therapeutic strategy [8,11,12,13] (see Table S1, Supplementary Materials). Given this global variation in guideline recommendations, this study aimed to assess real-world practice patterns regarding the post-cardioversion management of low-risk patients with acute AF via an international survey involving physicians and focusing on short-term OAC.

## 2. Materials and Methods

### 2.1. Survey Creation

**Figure 1:**
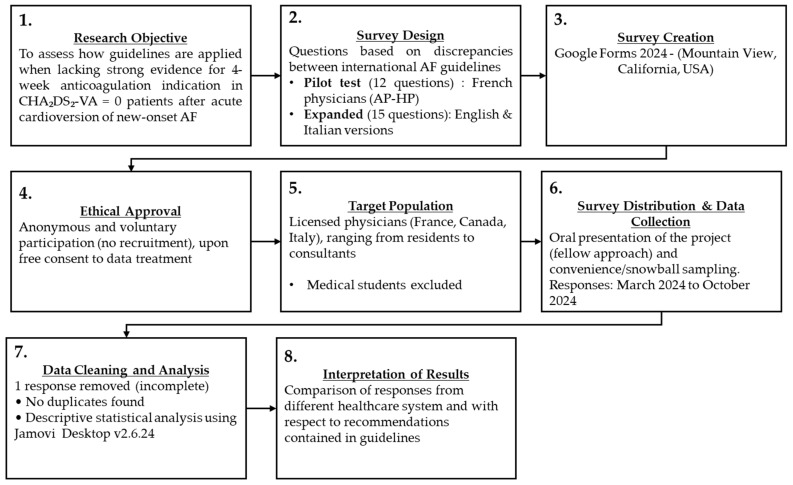
Study Design Flowchart. Description of the survey’s ideation, development, and distribution, illustrating the methodology and rationale behind this research survey. Abbreviations: AF (Atrial Fibrillation); AP-HP (Assistance Publique Hôpitaux de Paris, i.e., Parisian university hospital trust); OAC (Oral Anticoagulation).

PubMed/MEDLINE and Embase databases were used to identify the most relevant guidelines on AF via the following search phrase: “atrial fibrillation AND guidelines”. The following results, in English, dating from 2018 onwards were included for review: ESC 2024 (Europe) [11], China 2024 [10], USA 2023 [8], UK (NICE) 2021 [12], CCS 2020 (Canada) [9], and Australia and New Zealand 2018 [13] (see Table S1, Supplementary Materials). Only the most recent and updated guidelines were used as the basis for this study, to ensure alignment with current standards and the latest advances in the management of AF patients. A cross-sectional survey was designed at *Università degli Studi di Torino* based on a review of the evidence presented in the selected guidelines (see Figure 1: *Study Design Flowchart* and Table S1—Supplementary Materials). *Google Forms 2024* (Mountain View, CA, USA) was employed for the development of the questionnaire. An a priori power analysis was performed using G*Power (G*Power 3, Faul et al., Düsseldorf—Germany, 2007 [14]) to estimate the required sample size for a Chi-square test of independence. Assuming a large effect size (Cohen’s *w* = 0.5), an α-level of 0.05, and desired power (β) of 0.80, the analysis showed that a minimum total sample size of 52 participants was required to detect a statistically significant association between categorical variables. The rationale for employing a large effect size (*w* = 0.5) in the power analysis was justified based on strong, guideline-driven differences in expected clinical decision-making between Canada (where short-term OAC is recommended to all patients after cardioversion) and European countries (where it is instead optional), leading to dramatic variations in physicians’ practices. A standardized clinical case describing a 64-year-old male undergoing electrical cardioversion for AF diagnosed within the previous 12 h, without any other comorbidities or regular medications, was presented as the starting point of the survey, onto which all questions were based. The survey included questions regarding physician demographics and the clinical management of acutely cardioverted low-risk AF patients. The full text of the survey, including the introductory clinical case and the complete set of questions investigated, is available in the Supplementary Materials (pages 2–5).

### 2.2. Survey Administration

The survey was distributed in collaboration with the *University of British Columbia (UBC) Division of Cardiology* and *IRCCS Ospedale San Raffaele*. We surveyed physicians from 17 centres across 5 countries (Canada, Italy, France, Germany, and USA) on 2 continents (Figure S1, Supplementary Materials). The survey was open for participation between March 2024 and October 2024. Physicians with a wide range of experience, ranging from first-year residents to consultants, were included, while medical students were excluded. Convenience and snowball sampling were used. More specifically, participants were recruited with a *fellow approach*, in which the more experienced authors involved refrained from sharing the questionnaire through official channels (e.g., through official email databases) with their colleagues, in order to truly understand real-world therapeutic choices and to avoid the risk of methodologically influencing responses. Two countries (USA and Germany) were inadvertently involved in the study as the survey link was shared by participants with colleagues from these countries with an interest in this topic.

Formal ethical approval from an institutional review board or Ethics Committee was not obtained for the following reasons:

I. Participants were not actively recruited. Instead, they voluntarily chose to take part in the survey, upon reviewing and consenting to *Google Forms’* data usage disclaimer with respect to data collection, storage, and protection. A dedicated section at the beginning of the survey explicitly communicated this and included the following consent statement: “*By participating in this survey, respondents acknowledge and agree to the Google Forms Terms of Service and consent to the use of their responses solely for research purposes, in compliance with the EU General Data Protection Regulation (GDPR)*.” (See Supplementary Materials, page 2). Additionally, although *Google Forms* may be used to collect and process personal data, no sensitive information (e.g., names, dates of birth, email addresses, or postal addresses) was requested for the survey completion.
II. The authors developed and distributed the survey using Google Forms, thereby agreeing to its Terms of Service and Guidelines (see Supplementary Materials pages. 2–6; *Google Forms Policies and Guidelines*).
III. All authors were fully committed to adhering to these policies throughout the research process, according to the latest version of the Declaration of Helsinki.

For these reasons and since all data were completely anonymous and did not involve patients, but rather physicians on a voluntary basis (not recruited), Ethics Committee approval was not required.

All categorical variables are reported as the number of respondents and a percentage. Such variables were compared by contingency tables, while Chi-square (χ^2^) and Fisher’s Exact Tests were employed to study the strength of associations. All tests of significance were two-tailed, and a *p* < 0.05 was considered statistically significant within a 95% CI. Analysis was performed using Jamovi Desktop (version 2.6.24, JASP Project, Sydney, Australia).

## 3. Results

Seventy-six physicians answered the questionnaire between March 2024 and October 2024. Most of the respondents were cardiologists (n = 60, 78.9%), followed by clinicians working in internal medicine and intensivists (n = 8, 10.5% and n = 5, 6.5%, respectively). Two respondents (2.6%) specialised in geriatrics and only 1 (1.3%) in emergency medicine. The majority of the clinicians (n = 48, 63.2%) who replied to the question “*Would you start this patient on short-term OAC with DOACs*?” selected the option indicating short-term (4 weeks) OAC prescription following acute cardioversion in all patients, regardless of the CHA_2_DS_2_-VA score or AF duration. Six physicians (7.9%) would only anticoagulate patients with a CHA_2_DS_2_-VA score ≥ 1, while 11 participants (14.5%) reported that they would consider short-term OAC upon discussion with their patient (*shared decision-making* strategy) and further diagnostic workup. Another 11 practitioners (14.5%) decided not to prescribe any short-term OAC, believing that the patients’ haemorrhagic risk in the study population would outweigh the thromboembolic one. Starting temporary anticoagulation only in patients with a CHA_2_DS_2_-VA ≥ 1 was the least selected approach. Respondents’ replies, stratified by specialty and years of clinical experience, are summarised in Tables S2–S4 in the Supplementary Materials section. Although cardiologists mostly opted for proactive anticoagulation (cardiologists vs. other specialties, Fisher’s and χ^2^ tests *p*= 0.029, see Table S5, Supplementary Materials), they also represented the highest proportion of physicians who would avoid such treatment in low-risk patients after sinus rhythm restoration, with 9 of them (15% of all cardiologists) considering post-cardioversion OAC overtreatment.

### 3.1. Stratification by Work Experience

When considering expertise level, only 12 out of 41 participants (29.3%) with less than 5 years of clinical practice indicated that they would not initiate OAC after acute cardioversion. Those with more than 20 years of experience showed more divided opinions, with three cardiologists (from AP-HP, Milan, and Turin) stating that they would not prescribe short-term OAC after cardioversion to limit haemorrhagic complications, and 5 other cardiologists (1 from Turin and 4 from UBC) indicating that they would initiate anticoagulant therapy in all acutely cardioverted patients (see Tables S3 and S4, Supplementary Materials). Overall, younger physicians with less than 5 years of experience (n = 29/41, 70.7%) would prescribe short-term OAC more frequently compared to those with ≥ 10 years’ experience (n = 16/28, 57.1%, considering both those with more than 10 and more than 20 years of medical training). This latter association was further confirmed statistically by the Fisher’s Exact Test (*p* = 0.049, see Table 1).

### 3.2. Stratification by Healthcare System

**Figure 2:**
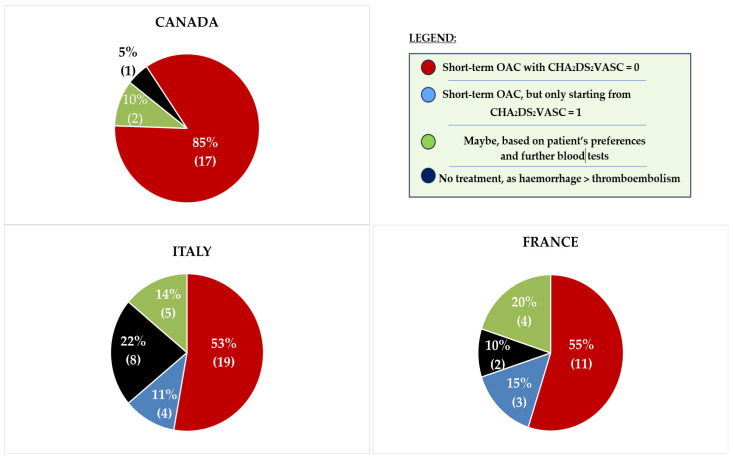
International differences in short-term OAC prescription. Note how the replies tend to be more varied in European countries, where short-term OAC after acute cardioversion is optional according to the ESC 2024 guidelines. In contrast, responses are more uniform in Canada, where OAC (unless absolute contraindications exist) is recommended to all patients undergoing cardioversion for at least 4 weeks, regardless of their thromboembolic risk score. All tables show a *p*-value < 0.01, determined by Chi-square test. Abbreviations: CHA_2_DS_2_-VA [C: Congestive heart failure; H: Hypertension; A_2_: Age 75 or older (2 points); D: Diabetes mellitus; S_2_: Stroke or transient ischemic attack (2 points); V: Vascular disease; A: Age 65 to 74]; OAC (Oral Anticoagulation); SDM (Shared Decision-Making).

Approximately 50% of European physicians indicated that they would prescribe short-term OAC in the scenario outlined in the questionnaire, while the other half either would not administer OAC or would consider it if CHA_2_DS_2_-VASc ≥ 1 (see Figure 2).

**Figure 3:**
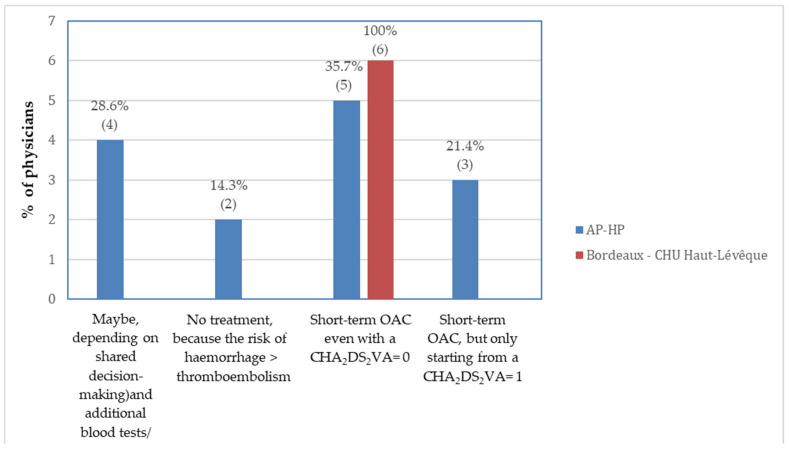
Differences in therapeutic decisions among French physicians. All physicians working in Bordeaux uniformly adopted an “anticoagulate all” approach, while respondents part of AP-HP (Parisian hospitals) chose various therapeutic schemes. Abbreviations: AP-HP (Assistance Publique—Hôpitaux de Paris); CHU (Centre Hospitalier Universitaire); OAC (Oral Anticoagulation).

When performing χ^2^ and Fisher’s tests to assess the strength of the association between healthcare system or nationality and the propensity to prescribe short-term OAC, no statistically significant relationship was found (*p* = 0.120, see Table 2). However, a “centre-specific” practice pattern was observed in two notable cases:

1. Turin had the lowest frequency of post-cardioversion OAC prescription compared to all other participating centres.

2. In Bordeaux, all clinicians unanimously selected the option “*Short-term OAC for four weeks for all patients, even with CHA_2_DS_2_-VASc score = 0*”, while responses from Parisian hospitals were more heterogeneous (Figure 3 and Table S4, Supplementary Materials).

A question that elicited particular heterogeneity among respondents concerned whether to administer Vitamin K Antagonist (VKA)-based OAC to patients with the same risk profile as the patient illustrated in the survey’s clinical vignette, but presenting instead with valvular AF (e.g., AF associated with moderate-severe mitral stenosis). In detail, 20% (n = 4) of French respondents, 27.8% (n = 10) of Italian physicians, and 9.5% (n = 2) of Canadians stated they would not initiate short-term VKA-based OAC in an acutely cardioverted, valvular AF patient. Other notable international differences emerged in the use of imaging techniques for low-risk patients prior to cardioversion and in rhythm monitoring strategies after successful restoration of sinus rhythm. While 81.8% (n = 27) of Italian and German participants indicated that they would perform transthoracic echocardiogram (TTE) before cardioversion, 85.7% (n = 6) of Canadian respondents stated they would not, with Fisher’s testing revealing a statistically significant association between participants’ nationality and the use of TTE before cardioversion (Europe vs. Canada, *p* ≤ 0.001, see Table S6, Supplementary Materials). As far as preprocedural transoesophageal echocardiogram (TOE) is concerned, opinions were more homogeneous, with only 25% (n = 5) of French, 16.7% (n = 6) of Italian and German, and 9.5% (n = 2) of North American practitioners affirming that they would always perform it prior to acute cardioversion, regardless of arrhythmia duration and patients’ thromboembolic risk factors. A statistically significant association emerged between different healthcare systems and peri-cardioversion TOE (Europe vs. Canada, *p* = 0.006, see Table S6, Supplementary Materials).

In Canada, the preferred rhythm monitoring strategy after successful cardioversion was a 24-h or 7-day ECG Holter performed at regular intervals before follow-up visits (option selected by 40% of physicians). In contrast, Italian participants were more likely to recommend that patients purchase a smartwatch capable of recording a single-lead ECG (45.7%, n = 16 doctors). In France, discontinuous rhythm monitoring, recording single ECGs at follow-up visits, was the most popular approach (46.7%, n = 7).

European and Canadian physicians also expressed differing perspectives regarding long-term rhythm control strategies. Approximately 45% (n = 15) of Italian physicians indicated that they would offer AF catheter ablation by means of pulmonary vein isolation (PVI) as a first-line treatment after sinus rhythm restoration in young and low-risk individuals, while 51.5% (n = 17) would propose it to their patients in case of AF recurrence and reduced quality of life. Although only 7 Canadians replied to this question, 85.7% (n = 6) would offer it in case of recurrent arrhythmia, and 14.3% (n = 1) would not consider it necessary in this specific case, suggesting that PVI may be offered more frequently by European cardiologists (Europe vs. Canada, *p* = 0.013; see Table S7, Supplementary Materials).

## 4. Discussion

### 4.1. Evidence Regarding Peri-Cardioversion OAC

Short-term OAC therapy during the 4 weeks following cardioversion is strongly recommended only in two countries: Canada and China [9,10]. Conversely, contrasting indications are reported in other guidelines (see Table S1, Supplementary Materials). Overall, guideline recommendations are generally weak in terms of both strength of benefit and quality of evidence (class IIb or *Weak Recommendation*; *Low-Quality Evidence*). This is primarily due to the reliance on observational studies as the basis for such recommendations. The most relevant and recent evidence derives from the Finnish CardioVersion (FinCV) study series [5,15,16], the Cleveland Clinic study [17], a study from the Danish National Patient Registry [18] and a study from the Emergency Department (ED) at Parma University Hospital [2]. The only prospective observational research is by Tampieri et al. [19], though its findings are limited by a small sample size (218 cardioversions). Unfortunately, these studies provide limited data on haemorrhagic complications [5,17], making it challenging to estimate short-term OAC-related haemorrhagic outcomes in these low-risk patients. Nonetheless, they report that most TECs tend to occur within the first 30 days following acute cardioversion [5,20]. While their incidence is limited (0% to 0.4%), TECs are lowest when the time to cardioversion is <12 h (0.2%) [15].

In general, it can be inferred that the uncertainty around a definite indication for postprocedural short-term OAC is likely related to the complex pathophysiology leading to an increased susceptibility to TECs in the weeks following cardioversion. Four main processes have been identified to explain this:

I. **Atrial stunning**, a temporary depression in left atrial appendage (LAA) mechanical function, resulting in decreased LAA emptying velocities, potentially favouring intracardiac thrombus formation [21]. The intensity of this phenomenon depends on the duration of the preceding AF episode and it can occur after both electrical and pharmacological cardioversion [4].
II. **An inaccurate estimation of the real onset of AF**. Up to 4–8% of patients whose AF lasts for just 6 to 48 h are completely asymptomatic [22], raising the risk of cardioversion being performed beyond the recommended 48-h window. This is clinically relevant, because longer AF episodes (even if asymptomatic) lead to more profound atrial stunning and a higher probability of post-cardioversion thrombus formation [22].
III. **Transient prothrombotic state associated with sinus rhythm restoration**. Even if AF lasts < 48 h, increased atrial volume and pressure can lead to atrial stretching and endothelial dysfunction, further promoting blood stasis and thrombin synthesis [23], both of which are risk factors for intracardiac thrombus formation.
IV. **Pre-formed thrombus within the left atrial appendage (LAA) or left atrium (LA)**. Although it has been demonstrated that intra-atrial thrombi can form even within 48 h of AF onset, [24], Anselmino et al. [25] detected no intracardiac thrombi in patients in sinus rhythm, with CHA_2_DS_2_-VASc = 0–1 and with no past history of AF ablation undergoing TOE before AF pulmonary vein isolation (PVI), making this hypothesis unlikely in the study population described in this research. Moreover, the absence of echocardiographic evidence of LAA thrombus does not guarantee safe cardioversion without appropriate postprocedural OAC, nor does it prevent TECs from occurring once sinus rhythm is restored [5].

Importantly, no statistically significant reduction in the rate of thromboembolic complications has been demonstrated upon administration of short-term OAC following acute cardioversion in low-risk patients [16,17], suggesting that OAC may be an overtreatment in this specific context [19]. However, as pointed out by Andrade et al., this lack of statistical significance is likely a consequence of the insufficient statistical power of subgroup analysis [26]. Still, two key elements could support postprocedural short-term OAC in this AF population: (1) uncertainty about precise AF duration and (2) patients’ unique clinical context and personal preferences. Indeed, asymptomatic paroxysmal AF patients could be mistakenly cardioverted well beyond the 12–48-h window recommended by guidelines [22], and without adequate OAC they might show a higher risk of embolic stroke following cardioversion, potentially resulting in devastating consequences in these otherwise healthy patients. Furthermore, the 30-day rate of thromboembolic events after acute cardioversion—even if performed within 12 h of AF onset—is approximately 0.2% [15]. Such incidence exceeds the monthly embolic event cut-off threshold of 0.12% (or 1.5% annual) employed by the Canadian Cardiovascular Society (CCS) to justify anticoagulation, therefore making OAC mandatory in this specific scenario, according to the 2020 CCS AF guidelines.

Nonetheless, as the bleeding risk is highest during the initial 30 days of OAC initiation [27], the ESC, AHA, Australian, and NICE (UK) indications recommend a more cautious and personalised assessment of the risks and benefits of this treatment, particularly in young patients (<65 years old) with no other comorbidities that can predispose them to TECs. Notably, Saglietto et al. estimated that, in a low-risk population (CHA_2_DS_2_-VASc = 0 and HAS-BLED = 0–1), the 30-day incidence of clinically significant bleeding following 4 weeks of OAC therapy after acute cardioversion would be approximately 0.46%. This includes a projected 0.08% risk of fatal intracranial haemorrhage (ICH), compared to a 0.2% risk of TECs if no OAC is prescribed [28].

A final point to consider is that AF guidelines are frequently applied in emergency room (ER) settings, where physicians can both perform cardioversion and prescribe short-term oral antithrombotic therapy. Hence, in this high-pressure context, a uniform approach that minimises reliance on precise AF onset timing and personalised management may be more practical and easier to adopt. In summary, as the Canadian guidelines wisely point out: “*The relative importance of a stroke prevented, and major bleed caused is a subjective judgement*” [9].

### 4.2. Study Results

The most striking finding of our study is the considerable variation in responses across both national and international participants when managing a common and straightforward clinical scenario. Key areas of discrepancy included: imaging techniques prior to cardioversion, the timing and type of rhythm monitoring, post-cardioversion anticoagulation, and the indication for first-line AF catheter ablation for long-term rhythm control. As the results of this survey demonstrate, some physicians may favour individualised strategies based on their own professional judgement and personal experience, even when these deviate from guideline recommendations. Although guidelines represent the standard of care, mirroring different local priorities and health needs and being designed on solid evidence, adherence was not always consistent. For instance, 15% (n = 3) of Canadian respondents reported not prescribing short-term OAC, despite national recommendations supporting its use after cardioversion in all patients. Furthermore, as illustrated in Figure 3, some centres demonstrated a highly consistent approach to post-cardioversion anticoagulation, while others—even within the same country and referring to the same guidelines—exhibited considerable variability. This highlights a significant degree of clinical discretion. Such inconsistency underscores the need for stronger and more uniform guideline recommendations to reduce disparities in patient management, which may otherwise lead to markedly different individual outcomes, even at the intranational level. A similar degree of heterogeneity regarding anticoagulation practices in the peri-cardioversion period was also observed in a prior EHRA survey [29], which shared some similarities with our questionnaire. However, that survey was conducted exclusively within Europe at a time when the CHA_2_DS_2_-VASc score was still in use; it was distributed using official email databases and was limited by a small sample size. It also did not offer any comparison of international AF guidelines and did not attempt to propose practical ways to tackle this *anticoagulation dilemma*.

Another remarkable aspect emerging from our study is that physicians with less than 5 years of clinical experience were more likely to prescribe OAC following cardioversion than their more experienced colleagues. This difference may be attributed to two key factors. First, younger physicians might be more familiar with and adhere more closely to the latest AF clinical guidelines given their more recent training and scientific education, together with easier access to digital tools and resources [30]. Consequently, they might tend to prioritise a *minimise stroke risk* approach, which is now a core principle emphasised in all AF guidelines. On the other hand, more experienced physicians in this study appeared to prefer a *case-by-case* strategy, carefully selecting patients who would benefit most from short-term OAC based on their professional judgement and experience, while also aiming to minimise haemorrhagic risk in these individuals. Overall, these findings highlight the important role that clinical experience and judgement play in shaping clinical decision-making, especially in those guideline “*grey areas*” in which recommendations permit more interpretation or where patients require a more individualised care.

However, caution is needed when interpreting these results. Since six association tests were conducted among distinct categorical variables, the possibility of type I errors (false positives) cannot be completely ruled out. In particular, the χ^2^ test examining the association between years of experience and OAC administration yielded a *p*-value of 0.049, which is just below the conventional significance value of 0.05. Furthermore, applying a Bonferroni correction for multiple comparisons (adjusted significance level α = 0.0083) would render this latter association, as well as the associations between specialty and OAC administration and between international differences in long-term rhythm control strategies, not statistically significant.

### 4.3. Future Implications

Overall, the diversity in participants’ responses in our survey reflects the need to further refine the guideline recommendations regarding short-term OAC after acute cardioversion to better orient and support clinical decision-making. We therefore propose three distinct evidence-based strategies that might be considered in the development of future recommendations, to optimise care for low-risk, acutely cardioverted AF patients:

(1) **Limiting the timing threshold for safe cardioversion without postprocedural OAC to <12 h**.

Acute AF cardioversion in patients with no risk factors for thromboembolism and not receiving postprocedural anticoagulation should be reduced to <12 h, as already recommended by the American AF guidelines [8]. The rationale for this suggestion derives from the FinCV studies [15], illustrating that only patients with CHA_2_DS_2_-VA = 0 and cardioverted within less than 12 h from AF onset truly carry a negligible post-cardioversion risk of thromboembolic complications (i.e., 0.2% within the first month that follows cardioversion). This 12-h limit is further supported by Sohara et al. [31], who showed that the transient prothrombotic state associated with AF becomes more intense with arrhythmia duration greater than 12 h, facilitating intracardiac thrombus formation.

(2) **Accounting for regional access, individual risk, and equity in prescribing OAC.**

Direct oral anticoagulants (DOACs) are to be preferred over Warfarin for short-term OAC after acute cardioversion in patients with nonvalvular AF, due to their lower propensity to cause intracranial haemorrhage (ICH) [32]. Furthermore, as the American and the Australian and New Zealand guidelines specifically mention, inequalities in access to DOACs and discriminations regarding anticoagulation should always be considered [8,13]. For instance, among Oceanian Aboriginals with AF, the risk of stroke of all types is three times higher than in the general population, particularly among younger individuals [13]. Additionally, such population resides in geographically isolated areas in which frequent INR monitoring for Warfarin therapy is impractical and DOACs are only accessible through out-of-pocket insurance, representing a relevant barrier to their use [13].

Although American guidelines are the only ones to explicitly address insurance coverage when selecting the optimal OAC treatment option for patients with AF, they also advocate that all patients should be equitably prescribed guideline-directed OAC, regardless of adverse social determinants of health, to minimise discrimination within the US healthcare system [8]. It is also pivotal to identify any other modifiable risk factors for TECs or bleeding not already part of CHA_2_DS_2_-VA or HAS-BLED, including drug interactions (e.g., ritonavir or other CYP inhibitors that might increase to dangerous levels plasmatic concentrations of DOACs) [32].

(3) **Implementing bleeding prevention strategies in systems with routine post-cardioversion OAC (e.g., Canada and China).**

While the CCS 2020 AF guidelines provide clear instructions on oral anticoagulation after cardioversion, they do not address patient education to minimise their risk of severe bleeding, which is potentially associated with short-term OAC. More precisely, acutely cardioverted, low-risk AF patients typically consist of relatively young (<65 years), active and otherwise healthy individuals. This group closely resembles that of individuals undergoing oral anticoagulation in the setting of venous thoracic outlet syndrome or after pulmonary embolism. In these contexts, it is recommended that high-risk physical activities be avoided while receiving OAC [33,34]. Similarly, while on OAC (i.e., for 4 weeks), low-risk, acutely cardioverted AF patients should refrain from taking part in any form of contact sports and risky behaviours or hobbies. In addition, cardioverted patients and their families should be educated on how to recognise early signs of severe bleeding and what to do in case such an event occurs.

## 5. Limitations

Although this study offers some valuable insights into real-world management practices after acute cardioversion of AF in the absence of globally adopted recommendations, its results should be interpreted in light of some limitations.

Firstly, the generalisability of our results is restricted. The practical strategies for improving patient outcomes and cardioversion safety described in this investigation are likely more applicable to cardiology departments than high-pressure acute care settings. This is primarily due to two main factors: (1) the relatively small sample size of survey participants and (2) the underrepresentation of Emergency Department (ED) physicians and general internists. Both these factors limit the applicability of our findings to real-world acute care settings, where rapid decision-making under time constraints, incomplete patient histories, and minimal physician–patient rapport are common. More in detail, cardiologists—who composed the vast majority of respondents—generally manage AF in more structured environments with easier access to diagnostic tools and longitudinal follow-up. Consequently, the therapeutic preferences emerging from this study may reflect an ideal management model of low-risk AF patients that is not always implementable in frontline clinical settings.

Secondly, an aspect which might further limit the widespread applicability of our results is the large effect size (*w* = 0.5) used for power analysis. While this choice was justified by substantial differences in AF management guidelines between Canadian and European healthcare systems, such a large effect size may have reduced the ability to detect more nuanced, but clinically important, differences in therapeutic strategies across specialties or healthcare systems following different guidelines. As a result, minor variations in decision-making (particularly in the ED or internal medicine settings) might have been overlooked, potentially leading to an overgeneralisation of cardiology-specific practices and primarily of routine anticoagulation after cardioversion.

All these limitations highlight the necessity for future studies including a broader and more balanced representation of physicians from multiple clinical environments. In the future, identifying the perspectives and strategies of ED physicians and internists will be of the utmost importance for developing common and general, yet adaptable, guidelines that account for the variability in diagnostic resources, time limitations, and patient characteristics encountered in non-specialist settings.

Despite these limitations, the international character of our study provides valuable insights into the variability of AF management. This degree of heterogeneity stresses the relevance of our findings and points to the necessity of ongoing efforts to harmonise care practices across healthcare systems.

## 6. Conclusions

Although based on a relatively small yet adequate sample reflecting cardiologists’ perspectives, this study highlights the clinical challenge of managing antithrombotic therapy in low-risk patients undergoing acute cardioversion for AF. The decision to initiate short-term oral anticoagulation (OAC) versus no therapy remains complex. Shortening the time window in which OAC can be safely withheld to less than 12 h, while accounting for access to DOACs, individual or regional stroke risk, and strategies to mitigate bleeding in those receiving short-term OAC, may reduce cardioversion-related complications. These considerations warrant integration into future clinical guidelines to optimise outcomes in this population.

## Figures and Tables

**Table 1:** Respondents’ choice regarding short-term oral anticoagulation (OAC), stratified by years of experience (<5 or >10 years). Chi-square and Fisher tests were performed and both results are statistically significant (*p* < 0.05). Note that participants with intermediate experience of between 5 and 10 years were excluded from this analysis. Abbreviations: DOAC (Direct Oral Anticoagulant); CHA_2_DS_2_-VA [C: Congestive heart failure; H: Hypertension; A_2_: Age 75 or older (2 points); D: Diabetes mellitus; S_2_: Stroke or transient ischemic attack (2 points); V: Vascular disease; A: Age 65 to 74]; Df (degrees of freedom).

	Would You Start This Patient on Short-Term (4 Weeks) DOAC Treatment?					
Years of Experience		Maybe, Based on Patient’s Preferences and Further Blood Tests	No Treatment Since the Haemorrhagic Risk > Thromboembolic Risk	Yes, for 4 Weeks but Only Starting from CHA_2_DS_2_-VA = 1	Yes, for 4 Weeks with CHA_2_DS_2_-VA = 0	Tot
<5	Observed	6	2	4	29	41
	% of total	8.7%	2.9%	5.8%	42.0%	59.4%
>10		3	8	1	16	28
		4.3%	11.6%	1.4%	23.2%	40.6%
Total		9	10	5	45	69
		13.0%	14.5%	7.2%	65.2%	100.0%
**Association Testing**						
	**Value**	**Df**	***p*** **-Value**			
χ^2^ Test	7.99	3	0.046			
Fisher’s Exact Test			0.049			
N	69					

**Table 2:** χ^2^ Test and Fisher tests to study the strength of the association between centre/nationality and the tendency to prescribe short-term OAC after acute cardioversion. No statistically significant relationship between centre of origin or belonging to a specific cardiology society and OAC prescription after cardioversion was identified (both tests’ *p* values > 0.05). DOAC: Direct Oral Anticoagulant.

	Centre		
Would You Start This Patient on Short-Term (4 Weeks) DOAC Treatment?	Canada	Europe	Tot
Maybe. Additional info and specific blood tests are required	2	9	11
No treatment since the haemorrhagic risk > thromboembolic risk	1	10	11
Yes, for 4 weeks but only starting from CHA_2_DS_2_VASc = 1	0	6	6
Yes, for 4 weeks with CHA_2_DS_2_VASc = 0	17	30	47
Total	20	55	75

Association Testing: χ^2^ Test: Value 6.50, Df = 3, *p* = 0.090. N = 75. Fisher’s Exact Test: *p* = 0.120. N = 75.

## Data Availability

All the data supporting the reported results can be found in the Supplementary Materials section.

## Supplementary Materials

The following supporting information can be downloaded at: https://www.mdpi.com/article/10.3390/medicina61071200/s1, Figure S1: Map illustrating the countries in which the survey was distributed; Table S1: Comparison of the specific recommendations regarding the indication for short-term post-cardioversion anticoagulation contained in different guidelines; Table S2: Choice of short-term oral anticoagulation (OAC), stratified by specialty and centre or nationality; Table S3: Choice of short-term oral anticoagulation (OAC), stratified by years of experience (<5, 5–10, >10, >20) and centre or nationality; Table S4: Choice of short-term oral anticoagulation (OAC), stratified by specialty, years of experience (<5, 5–10, >10, >20) and centres or nationality; Table S5: Chi^2^ and Fisher tests to assess the relationship between specialty and tendency to prescribe short-term OAC after acute cardioversion; Table S6: Chi^2^ and Fisher tests to study the association between nationality and TTE and TOE before acute cardioversion; Table S7: Chi^2^ and Fisher tests to study the association between nationality and first-line PVI in patients with newly diagnosed AF.

## Abbreviations

The following abbreviations are used in this manuscript:

AF	Atrial fibrillation
CV	Cardioversion
DOAC(s)	Direct oral anticoagulant(s)
OAC	Oral anticoagulation
TECs	Thromboembolic complications
