# Supplementary material for: Short-Term Anticoagulation After Cardioversion in New-Onset Atrial Fibrillation and Low Thromboembolic Risk: A Real-World International Investigation
Source: Medicina (Kaunas). 2025 Jun 30;61(7):1200. doi: 10.3390/medicina61071200 (PMC12299465; doi:10.3390/medicina61071200)
Supplement: Supplementary file 1 [file medicina-61-01200-s001.zip › medicina-3615688-supplementary.pdf]

SUPPLEMENTARY MATERIALS

TABLE S1. Comparison of the specific recommendations regarding the indication for short-term post-cardioversion anticoagulation contained in different guidelines.

NAME OF GUIDELINE & YEAR	CLASS & LEVEL OF EVIDENCE	RECOMMENDATION
**ESC – European Society of Cardiology 2024** [9]	I = Evidence and/or general agreement that a given treatment or procedure is beneficial, useful, effective.<br><br>B = Data derived from a single randomized clinical trial or large non-randomized studies.	"Only for those without thromboembolic risk factors and sinus rhythm restoration within 24h of AF onset is post-cardioversion OAC optional."<br><br>(p. 42)<br><br>"Oral anticoagulation is recommended to continue for at least 4 weeks in all patients after cardioversion and long-term in patients with thromboembolic risk factor(s) irrespective of whether sinus rhythm is achieved, to prevent thromboembolism."
**AHA/ACC/HRS Guidelines 2023 (USA)** [10]	IIb = benefit ≥ risk. May be reasonable/considered. Usefulness/effectiveness is unknown/unclear/uncertain or not well-established.<br><br>B-NR (nonrandomized) = moderate-quality of evidence from 1 or more well-designed, well-executed nonrandomized studies, observational studies or registry studies and metanalysis of such studies.	"In patients with low thromboembolic risks (CHA_2DS_2-VASc 0-1 or equivalent) and AF duration of <12 hours, the benefit of precardioversion imaging or pericardioversion anticoagulation is uncertain given the low incidence of pericardioversion thromboembolic events in this population." (p. e69)
**CCS – Canadian Cardiovascular Society 2020** [11]	Weak Recommendation;<br>Low-Quality Evidence	"We suggest that, in the absence of a strong contraindication, all patients who undergo cardioversion of AF receive at least 4 weeks of therapeutic anticoagulation adjusted-dose VKA or a DOAC) after cardioversion." (p. 1884)

**Chinese Guidelines 2023** [12]	IIb = Insufficient evidence/opinion to adequately support usefulness and efficacy. May be considered.<br><br>C = The evidence is based on consensus opinion of experts, case studies, or standard of care	"[…] the Guidelines recommend that for patients with AF duration of < 12 h and no recent history of stroke/transient ischemic attack, or for patients with AF duration 12–48h and low thromboembolic risk (CHA_2DS_2-VASc score = 0 for male or 1 for female patients), cardioversion without transesophageal echocardiography examination can be considered, while initiating oral anticoagulants therapy." (p. 268)
**NICE Guidelines, UK 2021** [7]	"In people with a confirmed diagnosis of atrial fibrillation of recent onset (less than 48 hours since onset), offer oral anticoagulation if:<br><br>1) stable sinus rhythm is not successfully restored within the same 48-hour period after onset of atrial fibrillation or<br><br>2) There are factors indicating a high risk of atrial fibrillation recurrence, including history of failed cardioversion, structural heart disease, prolonged atrial fibrillation (more than 12 months), or previous recurrences. or<br><br>3) it is recommended in section 1.2 on assessment of stroke and bleeding risks and section 1.6 on stroke prevention." [thromboembolic risk stratification using the CHA_2DS_2-VASc score] (1.8.9)	[Hence, it is possible to infer that patients with a CHA_2DS_2-VASc score ≤1 with an AF episode lasting < 48 hours can undergo electrical CV without any further need for anticoagulation during the 4 weeks following the procedure]
**Australian clinical guidelines** 2018 [8]	GRADE quality of Evidence: LOW; GRADE strength of recommendation: STRONG	"Anticoagulation is recommended at the time of electrical or pharmacological cardioversion, and for at least four weeks post-procedurally." (p. 1212)<br>From FIGURE 2 (p. 1223):<br>"No OAC necessary if AF duration is less than or equal to 48 hours."<br><br>• If CHA_2DS_2-VASc score = 0, "consider OAC for 4 weeks"<br><br>• If CHA_2DS_2-VASc score = 1, "consider OAC long term" [based on reassessment of CHA_2DS_2-VASc score]

Link to the survey – GOOGLE FORMS:
https://docs.google.com/forms/d/e/1FAIpQLSf2YzGCQn5HIr1KwjTrR-C6v1CHalVJQnz1NBOmfRF1FCiHGw/viewform?usp=sf_link

Google forms Policies and Guidelines: https://transparency.google/our-policies/product-terms/google-forms/

PRIVACY & DATA TREATMENT (see also: *Ethics Committee approval*, page 6)
By participating in this survey, respondents acknowledge and agree to the Google Forms Terms of Service and consent to the use of their responses solely for research purposes, in compliance with the EU General Data Protection Regulation (GDPR).

SURVEY'S FULL TEXT
Short-term anticoagulation after acute cardioversion of early-onset atrial fibrillation (<12-48h)
This survey's main goal is to understand the therapeutical choices of physicians in the absence of any international consensus. As of today, a lack of consistency exists between the European guidelines on atrial fibrillation (ESC 2024), the Canadian ones (CCS 2020) and the American ones (AHA et al. 2023) on whether short-term oral **anticoagulant therapy** for 4 weeks should be administered **following acute cardioversion** in patients with **early-onset atrial fibrillation (< 48h)** presenting with a **CHA2DS2VASC score equal to 0 or 1***.
After having read the following clinical case, answer the questions to the best of your knowledge.

CLINICAL CASE:
Mr. P., 64 years old, has just undergone electrical cardioversion for his atrial fibrillation **discovered 12 hours ago, following a first acute episode of palpitations.**
Past medical history: nothing worth mentioning. Mr P. does not smoke, but he is a moderate social drinker at family gatherings or after work meetings. He isn't on any medical treatment, nor does he take any NSAIDS on a regular basis.
Clinically: Following cardioversion, Mr P. is back to sinus rhythm (HR 87), BP 130/80, SpO2 99% with no need for supplemental O2. He is afebrile. Heart sounds are regular, with no perceivable heart murmurs. The patient is hemodynamically stable and does not present any sings of shock.
Blood tests: eGFR 70 mL/min/1.73m2, Hb 14 g/dL, pH 7.37, pCO2 40 mmHg, pO2 90 mmHg

* The newly released ESC 2024 guidelines recommend the use of the CHA_2DS_2-VA score, the sexless version of the CHA_2DS_2-VASc score, in order to stratify patients according to their risk of thromboembolism. However, in this specific clinical case, both scores can be interchangeably used, since the patient under study is a male.

This survey has been proposed in the following countries:

1. Italy (Università degli Studi di Torino, Milano and Roma)
2. France (AP-HP Paris, CHU Bordeaux Haut-Lévêque)
3. Canada (Vancouver General Hospital and Toronto General Hospital)

This research did not receive any specific grant from founding agencies in the public, commercial or not-for-profit sectors.

The following clinical questions composed the questionnaire:

1. What is your nationality/In which centre do you work?

2. Which is your specialty/what are you specializing in?

3. How long have you been working for?
   - Less than 5 years
   - 5 to 10 years
   - > 10 years
   - > 20 years

4. Are you trained and confident in performing both electrical and pharmacological cardioversions?
   - Yes, I am confident in performing both of them
   - No
   - Only the pharmacological one
   - Only the electrical one

5. Would you perform a TTE (trans-thoracic echocardiogram) in this patient prior to cardioversion (CV)? If yes, starting from which CHA2DS2VASC score do you regularly do?
   - No, no need for it, since this patient's atrial fibrillation was discovered just < 48 hours ago
   - Yes, regardless of the CHA2DS2VASC score
   - Yes, but starting from a score ≥ 1
   - Yes, but starting from a score = 2

6. Would you perform a TTE (trans-thoracic echocardiogram) in this patient prior to cardioversion?
   - Yes
   - No

7. Would you perform any periprocedural anticoagulation by NOAC/VKA/LMWH/UFH (loading dose)?
   - Yes
   - No

8. If you answered yes to the previous question, which protocol for periprocedural anticoagulation is used in your hospital? → open question

9. How would you monitor this patient for atrial fibrillation recurrences following cardioversion?
   - By providing the patient with a portable ECG recorder, to analyse his/her rhythm in case of symptoms
   - Discontinuous monitoring, performing ECGs during planned follow-up visits
   - 24h 7D Holter recording at frequent intervals, at least for the initial months following cardioversion
   - Continuous monitoring via loop recorder implantation

- I would advise my patient to buy a smartwatch or any other similar wristband with photoplethysmography, so to help in the diagnosis of a new episode of AF.

10. Which score would you use to assess the bleeding risk linked to anticoagulant therapy for this patient?
    - HAS-BLED
    - ATRIA
    - ORBIT

11. Would you start this patient on oral anticoagulant therapy with DOACs?
    - Yes, during 4 weeks with a CHA2DS2VASC = 0
    - Yes, during 4 weeks, but only strarting from a CHA2DS2VASC = 1
    - No, no need to treat this patient, since the haemorrhagic risk would outweigh the thromboembolic one
    - Maybe. In order to decide I would discuss with the patient about the benefits and the risks of OAC and I would run some blood tests (ex. checking his coagulation panel, AT, factor VII, troponin, BNP, growth differentiation factor-15)

12. If you answered YES to the previous question, how would you control the bleeding risk for a patient on DOAC treatment?
    - I would try to reduce the patient's risk factors for bleeding, such as hypertension, excess alcohol intake, intake of antiplatelets drugs & NSAIDS, anemia and renal /hepatic failure
    - I would instruct the patient on how to recognize acute signs and symptoms of haemorrhage, e.g. hypotension, "thunderclap" headache, double vision, abdominal pain following low-energy trauma
    - I would run regular blood tests to check for INR, aPTT, bleeding time, fibrinogen, ecc.
    - Altro:

13. Would you treat a patient with the same risk profile by AVK (Warfarin) if his/her atrial fibrillation were to be valvular (ex. associated with mitral stenosis), considering the higher risk of intracranial bleeding associated with it, when compared to NOAC?
    - Yes
    - No

14. Would you offer this patient transcatheter ablation (PVI) as a long-term rhythm control strategy?
    - Yes, in any case
    - Yes, but only in case of persistence of symptoms and reduced quality of life
    - No, that's not necessary

15. Would you suggest any other therapeutic or diagnostic strategy for this patient? → open question

NB: The initial survey included 12 items, both open-ended and multiple choice closed-ended questions. Following a pilot phase in France, 4 extra items were investigate to expand on the concepts present in the 12 question pilot.

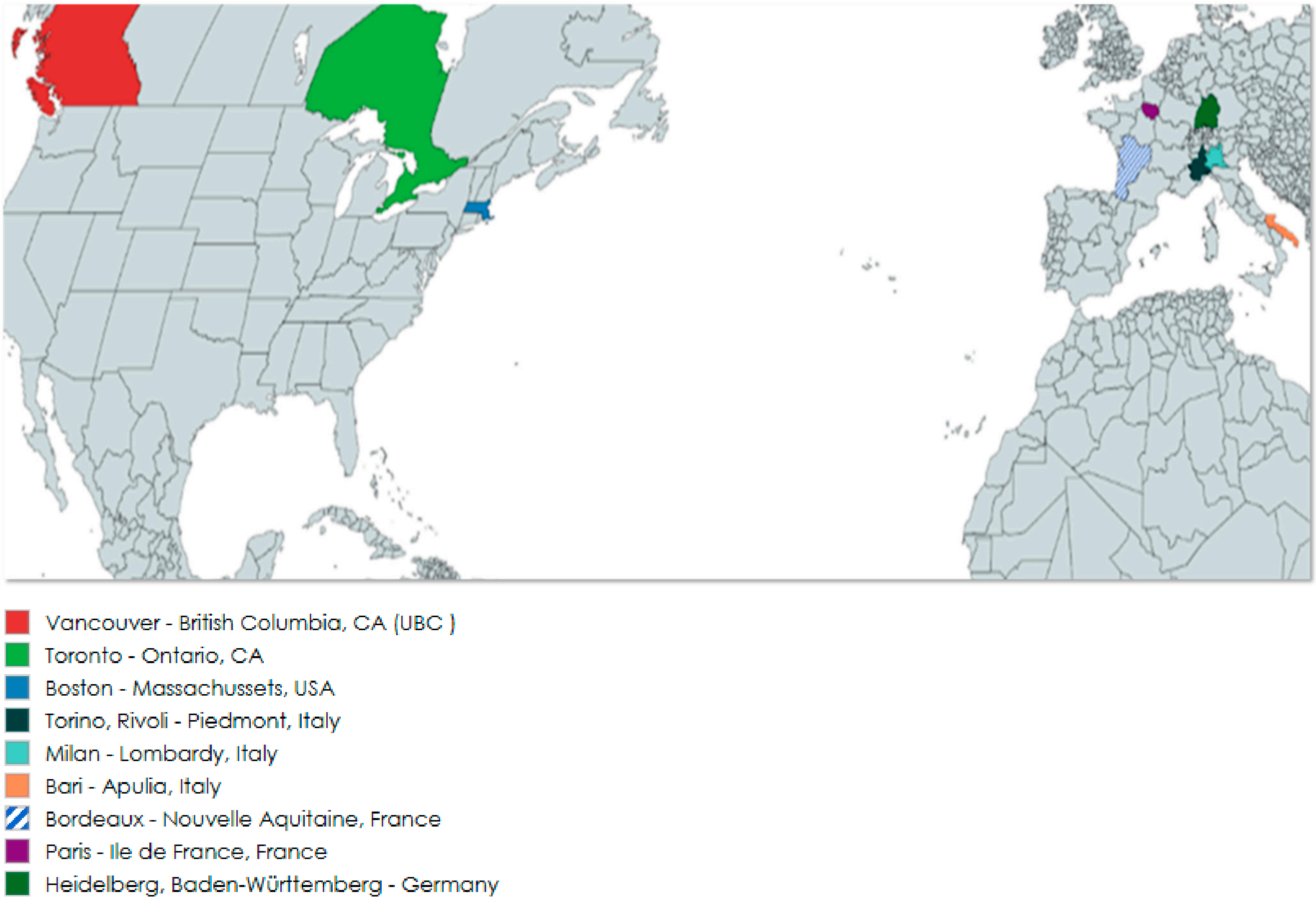

FIGURE S1. Map illustrating the countries in which the survey was distributed. In Canada, Italy and France a QR code for accessing the survey was manually distributed, while physicians in Germany and the US were inadvertently involved in this investigation, by receiving a link to the open-access survey from other physicians previously involved in this survey.

The QR code for accessing the survey on *Google Forms* was manually distributed in 17 major hospitals of 3 countries:

- **ITALY**:
    - Turin [AOU San Luigi Gonzaga, AOU Molinette - Città della Salute, Ospedale Mauriziano Umberto I],
    - Milan [IRCCS San Raffaele and ASST-GOM Niguarda],
    - Bari [Ospedale di Venere, Apulia]),
- **FRANCE**
    - AP-HP [Assistance Publique - Hôpitaux de Paris]
    - Bordeaux [CHU Haut – Lévêque],
- **CANADA**: Vancouver General Hospital and St. Paul's Hospital). No responses were obtained from Toronto General Hospital, although the survey was distributed there too.

The survey was completed between March 2024 and October 2024.

NB It is worth noting, that only 1 response from the US and 1 response from Germany were obtained; thus, such responses were grouped by geographical proximity for the statistical analysis of data (i.e. the one American participant was considered together with responses from Canada, while the one from Germany was grouped with respondents from Italy). Also, although some centres were involved and received a QR code for assessing the survey, no responses were obtained (e.g. Toronto General Hospital).

ETHICS COMMITTEE APPROVAL

In the present study no formal ethical approval was obtained from any specific institutional review board or ethics committee. This decision was based on the following rationale:

I. Participants were not actively recruited. Instead, they voluntarily chose to participate in the survey. In fact, based on study design it was not possible to identify or approach a specific ethics committee, as no direct institutional affiliation nor a particular organization could be established.

II. The authors developed and distributed the survey using Google Forms, thereby agreeing to its Terms of Service and Guidelines. All authors were fully committed to adhering to these policies throughout the research process, as to the latest version of the Declaration of Helsinki.

III. All participating physicians, by submitting their responses, consented to the data usage disclaimer, which detailed how data are collected, stored, and protected. A dedicated section at the beginning of the survey explicitly communicated this, and included the following consent statement:

"*By participating in this survey, respondents acknowledge and agree to the Google Forms Terms of Service and consent to the use of their responses solely for research purposes, in compliance with the EU General Data Protection Regulation (GDPR).*", see Supplementary Materials (page 2).

IV. Although Google Forms may be used to collect and process personal data, no sensitive information—such as names, dates of birth, email addresses, or postal addresses—was requested in the survey.

SHORT-TERM OAC PRESCRIPTION (4 WEEKS)	AP-HP (FRA)	Bari – Osp. Di Venere (ITA)	Bordeaux - CHU Haut-Lévêque (FRA)	Milan – Niguarda (ITA)	Milan - S. Raffaele (ITA)	Turin Uni (ITA)	UBC cardio (CAN)	UBC cardio (AUS)	Heidelberg – Universitätsklinikum (GER)	Boston (USA)	TOT
**Maybe. Additional info and specific blood tests are required.**	**4**	**1**			**3**	**1**	**2**				**11**
Cardio	2	1			2	1	2				8
Emergency med	1										1
ICU	1										1
Internal med					1						1
**No treatment since the haemorrhagic > thromboembolic risk**	**2**			**1**	**2**	**5**	**1**				**11**
Cardio	1			1	1	5	1				9
ICU	1										1
Internal med					1						1
**Yes, but only starting from a $CHA_2DS_2VASC = 1$**	**3**				**2**	**1**					**6**
Cardio					1	1					2
ICU	3										3
Internal med					1						1
**Yes, even with a $CHA_2DS_2VASC = 0$**	**5**	**1**	**6**	**3**	**9**	**5**	**16**	**1**	**1**	**1**	**48**
Cardio	2	1	5	3	5	5	13		1		35
Cardio EP					1		2	1			4
Geriatrics	1		1								2
Heart Transplant							1				1
Internal med	2				3						5
Interventional cardio										1	1
**TOT**	**14**	**2**	**6**	**4**	**16**	**12**	**19**	**1**	**1**	**1**	**76**

TABLE S2. Choice of short-term oral anticoagulation (OAC), stratified by specialty and centre or nationality. ICU (Intensive Care Unit). AP-HP (Assistance Publique – Hôpitaux de Paris), CHU (Centre Hospitalier Universitaire).

SHORT-TERM OAC PRESCRIPTION (4 WEEKS)	AP-HP (FRA)	Bari – Osp. Di Venere (ITA)	Bordeaux - CHU Haut-Lévêque (FRA)	Milan – Niguarda (ITA)	Milan - S. Raffaele (ITA)	Turin (ITA)	UBC cardio (CAN)	UBC cardio (AUS)	Heidelberg – Universitätsklinikum (GER)	Boston (USA)	TOT
**Maybe. Additional info and specific blood tests are required.**	4	1			3	1	2				11
< 5	4	1			1						6
> 10					1						1
> 20						1	1				2
5 to 10					1		1				2
**No treatment since the haemorrhagic risk > thromboembolic risk**	2			1	2	5	1				11
< 5					1	1					2
> 10				1		3	1				5
> 20	1				1	1					3
5 to 10	1										1
**Yes, during 4 weeks but only starting from CHA2DS2VASC = 1**	3				2	1					6
< 5	2				1	1					4
> 10					1						1
5 to 10	1										1
**Yes, during 4 weeks with CHA2DS2VASC = 0**	5	1	6	3	9	5	16	1	1	1	48
< 5	4	1	5	2	6	3	7		1		29
> 10	1			1	1	1	5	1			10
> 20						1	4			1	6
5 to 10			1		2						3
TOT	14	2	6	4	16	12	19	1	1	1	76

TABLE S3. Choice of short-term oral anticoagulation (OAC), stratified by years of experience (<5, 5-10, > 10, > 20) and centre or nationality.

	AP-HP	Bari (Ospedale Di Venere)	Bordeaux - CHU Haut-Lévêque	Milano (ASST GOM Niguarda)	Milano (S. Raffaele)	Turin	UBC cardiology	UBC cardiology (AUS)	Universitätsklinikum Heidelberg	USA (Boston)	TOT
Maybe. Additional info and specific blood tests are required.	**4**	**1**			**3**	**1**	**2**				**11**
**< 5**	**4**	**1**			**1**						**6**
Cardio	2	1									3
Emergency med	1										1
ICU	1										1
Internal med					1						1
**> 10**					**1**						**1**
Cardio					1						1
**> 20**						**1**	**1**				**2**
Cardio						1	1				2
**5 to 10**					**1**		**1**				**2**
Cardio					1		1				2
No treatment since the haemorrhagic risk > thromboembolic risk	**2**			**1**	**2**	**5**	**1**				**11**
**< 5**					**1**	**1**					**2**
Cardio						1					1
Internal med					1						1
**> 10**				**1**		**3**	**1**				**5**
Cardio				1		3	1				5
**> 20**	**1**				**1**	**1**					**3**
Cardio	1				1	1					3
**5 to 10**	**1**										**1**
ICU	1										1
Yes, during 4 weeks but only starting from CHA2DS2VASC = 1	**3**				**2**	**1**					**6**
**< 5**	**2**				**1**	**1**					**4**
Cardio						1					1
ICU	2										2
Internal med					1						1
**> 10**					***1***						***1***
Cardio					1						1
**5 to 10**	**1**										**1**
ICU	1										1
Yes, during 4 weeks with CHA2DS2VASC = 0	**5**	**1**	**6**	**3**	**9**	**5**	**16**	**1**	**1**	**1**	**48**
**< 5**	**4**	**1**	**5**	**2**	**6**	**3**	**7**		**1**		**29**
Cardio	1	1	4	2	3	3	6		1		21
Cardiology EP					1		1				2
Geriatrics	1		1								2
Internal med	2				2						4
**> 10**	**1**			**1**	**1**	**1**	**5**	**1**			**10**
Cardio	1			1	1	1	3				7
Cardiology EP							1	1			2
Heart Transplant							1				1
**> 20**						**1**	**4**			**1**	**6**
Cardio						1	4				5
Interventional cardio										1	1
**5 to 10**			**1**		**2**						**3**
Cardio			1		1						2
Internal med					1						1
**TOT**	**14**	**2**	**6**	**4**	**16**	**12**	**19**	**1**	**1**	**1**	**76**

TABLE S4. Choice of short-term oral anticoagulation (OAC), stratified by specialty, years of experience (<5, 5-10, > 10, > 20) and centres or nationality.

STATISTICAL ANALYSIS

Would you start short-term DOAC Tx?	Specialty		Total
	Cardio	Other	
Maybe. Additional info and specific blood tests are required.	8	3	11
No treatment since the haemorrhagic risk > thromboembolic risk	9	2	11
Yes, during 4 weeks but only starting from $CHA_2DS_2VASc = 1$	2	4	6
Yes, during 4 weeks with $CHA_2DS_2VASc = 0$	41	7	48
Total	60	16	76

χ^2 Tests

	Value	Df	p
χ^2	9.03	3	0.029
**Fisher's exact test**			0.029
**N**	76		

TABLE S5. **Chi^2 and Fisher tests** to assess the relationship between specialty and tendency to prescribe short-term OAC after acute cardioversion. A strong association between the two is clearly visible, with cardiologists being the most likely to adopt a "try to prevent thromboembolism at all costs" approach.

TTE prior to cardioversion	Centre		Total
	Canada	Europe	
No	19	6	25
Yes	1	27	28
Total	20	33	53

χ^2 Tests

	Value	df	p
**χ^2**	29.5	1	<.001
**Fisher's exact test**			<.001
**N**	53		

TOE in this patient prior to cardioversion	Centre		Total
	Canada	Europe	
No	17	37	54
Yes, but starting from a score = 2	0	4	4
Yes, but starting from a score = 2 in men	3	0	3
Yes, regardless of score	0	11	11
Yes, starting from a score ≥ 1	0	3	3
Yes, starting from a score ≥ 1 in men	0	1	1
Total	20	56	76

χ^2 Tests

	Value	df	p
χ^2	15.9	5	0.007
**Fisher's exact test**			0.006
**N**	76		

TABLE S6. **Chi^2 and Fisher tests** to study the association between **nationality and TTE and TOE before acute cardioversion**. In both cases, European physicians would perform imaging tests more frequently compared to Canadians.

Centre	Would you offer PVI: No	Yes, always	Yes, if symptoms persist	Tot
Canada	2	0	6	8
Europe	1	15	17	33
Total	3	15	23	41

χ^2 Tests

	Value	df	p
χ^2	8.52	2	0.014
**Fisher's exact test**			0.013
**N**	41		

TABLE S7. **Chi^2 and Fisher tests** to study the association between **nationality and first-line PVI in patients with newly diagnosed AF.** Europeans are shown to offer PVI more easily compared to Canadians (p= 0.013).
